# A New Vision for Clinical Trials in Africa

**DOI:** 10.1371/journal.pmed.0010071

**Published:** 2004-12-28

**Authors:** The PLoS Medicine Editors

## Abstract

A new funding body (the EDCTP) will fund clinical trials in developing countries, particularly in Africa, that help to develop affordable interventions against HIV, TB, and malaria. How is it doing so far?

## Teaser

A promising European funding body is stumbling over the details

Last year the European Parliament and Council formed the european and Developing Countries Clinical Trials Partnership (EDCTP). The aim of this new funding body, which has a budget of &euro;400 million spread over five years, is a noble one: to fund research in developing countries, particularly in Africa, that contributes to the development of affordable prophylactics and drugs for HIV/AIDS, tuberculosis, and malaria.

Unfortunately, the organization has not got off to an auspicious start. Its executive director, Piero Olliaro, was ousted from power at the first EDCTP annual forum at the end of September. There have been rumblings of discontent among grant applicants who say that the first round of grant assessments was administered poorly. And not-for-profit organizations that would like to partner with EDCTP have been left in the dark regarding whom to speak to at the organization.

This omission is significant because partnership is one of the key tenets of the EDCTP. European research agencies are slowly beginning to realize that they need to cooperate with each other if they are to be competitive with the United States. The history of many European countries is such that Europe has much stronger ties with Africa than does the United States, so it makes political sense for the European Union to fund research that provides a springboard for European researchers to compete effectively with US scientists.

Crucially, the EDCTP was also set up to enable European and African scientists to work together as equal partners. There is increasing recognition that the paternalistic, colonial attitude that pervaded “tropical medicine” in the past just will not do. The EDCTP hoped to change that by having a Partnership Board that contains equal numbers of African and European representatives. However, the EDCTP Assembly, which contains a representative from each of 14 EU member states but none from African countries, has the power to veto the decisions of the Partnership Board, which is supposedly the scientific decision-making authority.

Doing clinical trials in Africa is far from easy. There are too few adequately resourced research centers, and those that do consistently perform well are oversubscribed. Therefore, there is a clear need for "capacity building"—development of a research infrastructure, in terms of both equipment and personnel, that is capable of coping with the challenges of clinical trials. The EDCTP hopes to contribute to this essential endeavor by funding clinical trials that are sustainable in the long term. In particular, it believes that the best way to train a new generation of African scientists is by teaching them on the job, that is, involving them fully in the planning and execution of the trial, rather than flying in European experts who leave as soon as the trial is finished.

A commitment from European researchers to be engaged for the long term is essential for the success of these projects. In addition, partnerships need to be brokered with national programs in Africa to ensure that the new capacity can be sustained over time. The end goal is to produce centers of excellence that are run by Africans doing internationally recognized research that conforms to Good Clinical Practice guidelines. But this will only happen if African researchers are treated as equal partners and are allowed to be fully engaged in the projects that are taking place in their countries.

So can the EDCTP work, or is it doomed to failure? In many ways the organization has a great deal going for it. Although the budget of 3400 million spread over five years is tiny considering the combined burden of HIV/AIDS, tuberculosis, and malaria, it is important to remember that it is the biggest single European project for clinical trials in Africa. In many ways the EDCTP is a demonstration project: if some success can be achieved it is very likely that additional funds will follow. The project is certainly strengthened by the involvement of Pascoal Mocumbi, the former prime minister of Mozambique, as High Representative of the project. Mocumbi is highly respected by the global-health community and carries considerable weight with African politicians. Mobilization of political will within Africa will be essential if research capacity is to be sustained for the long term.

On the downside, it seems clear to most insiders that the management structure needs to be radically changed and partnership with other organizations needs to be improved. The EDCTP Assembly met on October 28 and 29 to discuss these issues and to elect a new leader. At the time this editorial went to press, there was still no public announcement of the outcome of this meeting. In addition, the political infighting that pervades European politics at all levels needs to be controlled, or at least managed effectively. This might be a tall order, but it is essential if this worthwhile and high-profile project is to succeed.

